# Locoregional Recurrence after Biochemical Incomplete Response in Differentiated Thyroid Cancer Patients: Insights into Influencing Clinicopathological Factors and the Potential Role of ^18^ F-FDG PET/CT

**DOI:** 10.1055/s-0045-1806800

**Published:** 2025-03-24

**Authors:** Mai Amr Elahmadawy, Ismail Mohamed Ali, Ibrahim Mansour Nasr, Omnia Talaat

**Affiliations:** 1 Nuclear Medicine Unit, National Cancer Institute (NCI), Cairo University, Cairo, Egypt; 2 Radiodiagnosis Department, Faculty of Medicine, Zagazig University, Zagazig, Egypt; 3 Clinical Oncology and Nuclear Medicine Department, Faculty of Medicine, Zagazig University, Zagazig, Egypt

**Keywords:** BIR, thyroid cancer, locoregional recurrence, Tg, PET/CT

## Abstract

**Objective:**

The aim of this study was to evaluate the clinicopathological factors and stimulated thyroglobulin (Tg) course related to the occurrence of locoregional recurrence (LRR) in differentiated thyroid cancer (DTC) patients with biochemical incomplete response (BIR) as well as the value of fluorine-18 fluorodeoxyglucose ( ^18^ F-FDG) positron emission tomography (PET)/computed tomography (CT) in these patients.

**Methods:**

A total of 253 DTC adult patients initially treated with total thyroidectomy and iodine-131 (RAI-131) ablation and showed BIR on follow-up were enrolled in the study. All clinical, laboratory, pathological, radiological, and follow-up data were retrieved from their records.

**Results:**

Seventy-three out of the 253 BIR patients developed LRR during follow-up with the median time to recurrence of 27 months. In all, 61.6% of those who developed LRR were females, 78.1% were papillary thyroid carcinomas, 35.6% had initial regional nodal deposits, and the primary tumors were T2 and T3 in 78.2% of these patients ( *p* < 0.05). The median Tg level for those who developed LRR compared with those who remained free was 18 versus 17 ng/mL, respectively, at 6 months of follow-up. Meanwhile, on further 1-year follow-up, the median value spontaneously increased for the positive group to 37.6 ng/mL and decreased for those who remained free to 3 ng/mL. Eighty percent of the patients with a rising course of Tg level developed structural LRR ( *p* < 0.001). ^18^ F-FDG PET/CT showed the highest sensitivity and negative predictive value (NPV) of 100% in the detection of LRR compared with sensitivity values of 92.86 and 85.71% and NPV of 99.58 and 99.17%, respectively, with ultrasound (US) and RAI-131 scan. Meanwhile the highest specificity and positive predictive value (PPV) of 100% were observed noted with RAI-131 compared to specificity values of 99.16 and 99.58% and PPV of 87.50 and 92.86% with PET/CT and US, respectively. A cutoff point (SUVmax of 3.65) was successfully marked to discriminate those positive versus negative LRR with sensitivity and specificity of 100% and *p* -value of less than 0.001.

**Conclusion:**

Structural LRR after BIR appears to be more commonly associated with worse clinicopathological parameters and the incremental Tg levels serve as an indicator of its higher incidence. ^18^ F-FDG PET/CT has been shown to be a valuable diagnostic tool rather than a prognostic one in these patients.

## Introduction

The management of differentiated thyroid cancer (DTC) has undergone significant changes over the past decades, driven by large-scale biological studies, improved diagnostic tools, and therapeutic advances. 1

Initial risk stratification constitutes a starting point that guides initial management and follow-up but represents a static picture that does not consider response to initial treatment. 2 Hence, Tuttle and Alzahrani proposed the dynamic risk assessment (DRA) based on new integrated data that become available during the initial follow-up. 3 Such a strategy has been well received and has been adopted by several societies. 4 5 6

The DRA approach involves restratification of the initial risk assessment of DTC patients taking into account different responses to treatment: excellent, indeterminate, biochemical incomplete response (BIR), and structural incomplete response (SIR). This is done using data obtained during follow-up: thyroglobulin (Tg) and anti-Tg values, imaging techniques, including neck ultrasound (US), radioactive iodine-131 (RAI-131) whole-body scans, computed tomography (CT), and fluorine-18 fluorodeoxyglucose positron emission tomography CT ( ^18^ F-FDG PET/CT). 2

BIR is not an uncommon event after initial treatment with reported prevalence varying from 11 to 22%. BIR encompasses persistent elevated Tg values or anti-Tg antibodies (TgAb) without evidence of structural disease. 7 The incidence of developing structural recurrence varies across published data and the outcomes of such patients remain quite heterogeneous. A comprehensive data analysis of the factors linked to clinical outcome in this scenario is not adequately addressed. 8

^18^ F-FDG PET/CT scan has an established role in the detection of local and distant recurrence in DTC patients, especially when the Tg level is elevated and other imaging modalities are negative. 9 Also, the magnitude of the metabolic activity may reflect disease aggressiveness, and thus can provide information about the long-term outcome. 10 Therefore, the findings provided by ^18^ F-FDG PET/CT might modify the therapeutic approach in up to 30% of patients. 11 The present study aims at assessing the relation of various clinicopathological factors with the occurrence of locoregional recurrence in DTC patients with BIR, the role of posttreatment stimulated Tg in the dynamic assessment of such patients, and the performance of ^18^ F-FDG PET/CT in the detection and prediction of outcome of structural recurrence versus standard techniques.

## Patients and Methods

### Study Population

In total, 2,000 files for DTC patients who have received radioactive iodine therapy at the Nuclear Medicine Unit, National Cancer Institute (NCI), Cairo University, were reviewed in the period from 2000 to 2021. A total of 253 DTC adult patients who were initially treated with total thyroidectomy and RAI-131 ablation and showed BIR on follow-up were enrolled in the study. All demographic, clinical, laboratory, pathological, radiological, and follow-up data were retrieved from their records. All methods were performed in accordance with relevant guidelines and regulations. The study was approved by the Ethical Committee at NCI, with Institutional Review Board (IRB) number RO2311–309–062. Informed consent was waived due to the retrospective nature of the study and the analysis used anonymized clinical data.

The patients were stratified using the eighth edition of the American Joint Committee on Cancer/International Union against Cancer (AJCC/UICC) staging system. 12 Fixed doses of RAI-131 ablation were given (range: 30–100 mCi). The response assessment was evaluated at 6 and 12 months after RAI-131 therapy and at last follow-up. The follow-up included serum Tg assessment, neck US, and RAI whole-body scan. Neck US was performed by an experienced radiologist, and RAI-131 whole-body scan was performed 4 to 7 days after therapeutic RAI-131 and 2 days after diagnostic RAI-131 and was interpreted by an experienced nuclear physician. ^18^ F-FDG PET/CT was used during follow-up to detect structural recurrent disease in patients who showed BIR.

Therapy response was categorized according to the American Thyroid Association (ATA) management guidelines into excellent response (ER), indeterminate response (IR), BIR, and SIR. The patients were divided into a complete response group and an incomplete response group (including patients showing IR, BIR, or SIR) according to the response to therapy for the purpose of predictive correlations with response to therapy. 13

Locoregional structural recurrence was defined as tumor in the operative bed or regional lymph nodes detected by RAI-131, US, and/or ^18^ F-FDG PET/CT and proven histologically, after a documented tumor-free period.

### Imaging Techniques and Diagnostic Procedures

#### RAI-131 Therapy

One to 2 weeks prior to the 131-I therapy, the patient begins a low-iodine diet. Radioiodine therapy was given after T4 withdrawal for approximately 4 weeks to ensure the thyroid-stimulating hormone (TSH) level was greater than 30 mU/L, which was measured at therapy day in association with measuring the serum Tg level. Patients were hospitalized for 3 to 5 days according to the therapy dose given. T4 treatment was resumed on the fourth day with a posttherapy whole-body scan performed 5 to 7 days after oral intake of I-131.

In between therapy doses, patients were maintained on thyroxine suppressive therapy to keep the TSH level around 0.01 mU/L.

#### Serum Thyroglobulin Measurement

Serum Tg was assessed in our institute's laboratory using one commercial immunometric assay, and the same assay was used throughout a patient's follow-up. Stimulated Tg (TSH ≥30 mU/L after thyroid hormone withdrawal) was evaluated in the current study.

#### Neck Ultrasound

Neck US was performed in all patients by experienced sonographers using high-resolution US linear array transducer (7–15 MHz). The patients were placed in the supine position with the neck hyperextended. Any suspicious operative bed lesions or regional lymph nodes (>10 mm) were reported with their characteristics including site and size.

### ^18^ F-FDG PET/CT Protocol and Interpretation

^18^ F-FDG PET/CT study was performed using a Discovery PET/CT scanner (GE Medical System, Milwaukee, WI, United States). The study protocol was performed in accordance with the European Association of Nuclear Medicine (EANM) procedure guidelines for tumor imaging, version 2.0. 14 Images were reconstructed according to our departmental protocol.

^18^ F-FDG PET/CT images were reviewed and analyzed on the manufacturer's GE review station, which provided multiplanar reformatted images and enabled the display of the PET images, CT images, and fused PET/CT images. The images were interpreted by an experienced nuclear medicine physician.

Positive scan findings were considered when abnormal/nonphysiological focal FDG uptake was noted at PET images above the background activity. The spherical volume of interest (VOI) was drawn over the regions of interest (operative bed or regional nodal metastases) and the maximum standardized uptake value (SUVmax) was recorded. The whole-body images were also evaluated for distant sites of metastases.

### Statistical Analysis

Data were coded and entered using the Statistical Package for the Social Sciences (SPSS) version 28 (IBM Corp., Armonk, NY, United States). Data were summarized using mean, standard deviation, median, minimum and maximum in quantitative data, and frequency (count) and relative frequency (percentage) for categorical data. Comparisons between quantitative variables were done using the nonparametric Kruskal–Wallis and Mann–Whitney tests. 15 For comparing categorical data, the chi-squared ( *χ* ^2^ ) test was performed. The exact test was used instead when the expected frequency was less than 5. 16 Standard diagnostic indices including sensitivity, specificity, positive predictive value (PPV), negative predictive value (NPV), and diagnostic efficacy were calculated as described by Galen. 17 The receiver operating characteristic (ROC) curve was constructed with area under curve analysis performed to detect the best cutoff value of Tg and SUV for the detection of different outcomes. A *p* -value less than 0.05 was considered statistically significant.

## Results

### Biochemical Incomplete Response

This study enrolled 253 DTC patients treated with surgery and RAI-131 ablation and showed BIR. Females comprised 184 (72.7%) patients in the study group and 69 (27.3%) were males, with the female-to-male ratio of 2.7:1.

Their median age was 43 (range: 19–82) years. In all, 108 patients (42.7%) were ≥45 years. Papillary thyroid carcinoma (PTC) was the predominant pathology, representing 85.4% of the group, followed by follicular thyroid carcinoma (FTC) at 11.1%, and the Hürthle cell at 3.6%. The median size of the primary tumor was 2.4 cm (range, 0.8–9 cm). The initial staging was T1 in 115 (45.45%) patients, T2 in 88 (34.78%) patients, and T3 in 50 (19.76%) patients. In all, 68 (26.9%) patients were classified as N1, and all of them were M0. The capsular invasion was positive in 57 patients (22.5%). The vascular invasion was positive in 21 (8.3%) patients.

Initial management of the enrolled group was surgery, followed by RAI-131 ablation. Surgery was total thyroidectomy in 203 patients (80.2% of the group) and was total thyroidectomy with lymphadenectomy in the remaining 50 patients (19.8% of the group). After initial radio-surgical management, all patients started thyroxine suppressive therapy. All the patients revealed complete structural response with no radiologically detectable gross residual tumor, but an incomplete biochemical response, where the median value of the Tg level—while off Eltroxin—was 17 (range: 10–93) 6 months postablation. An additional 1-year follow-up revealed a gradual rise of Tg in 96 patients (37.9%) of the group; 148 (58.5%) patients had declining levels and 9 (3.6%) had stable Tg levels.

The median follow-up period was 76 months (range, 13–123 months). During follow-up, 73 patients developed locoregional recurrence and 7 (2.8%) patients developed distant metastases, 2 in the bone, 4 in the lung, and 1 in the brain. Thirteen patients (5.1%) died during follow-up.

### Locoregional Structural Recurrence after BIR

**Fig. 1:**
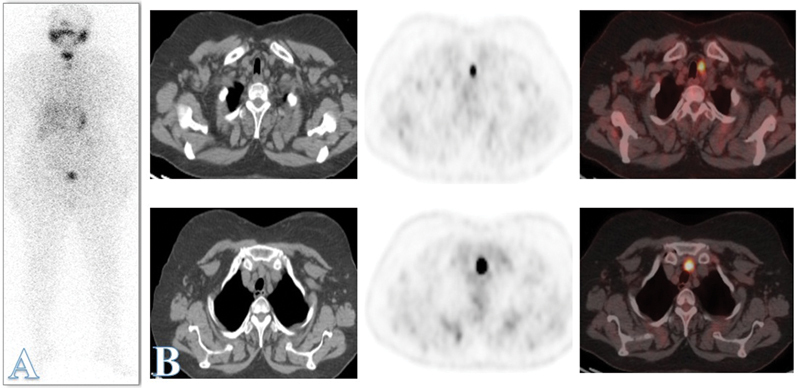
A 60-year-old female patient with papillary thyroid carcinoma underwent total thyroidectomy and received 100 mCi of RAI-131 for ablation followed by thyroxine suppressive therapy. At 6 months of follow-up, her stimulated thyroglobulin (Tg) level was 13 ng/mL with no evidence of any structural disease residue/recurrence on diagnostic RAI-131 whole-body scan or neck ultrasound (US) or computed tomography (CT) chest. ( **A** ) On further 3 months of follow-up, the stimulated Tg level showed further elevation to 27 ng/mL; thus, RAI-131 therapy was recommended, and posttherapy scan revealed Iodine uptake at thyroid operative bed and a smaller less active focal lesion is seen at the upper chest region likely nodal lesion. Neck ultrasound (US) revealed operative bed scarring and left paratracheal hypoechoic nodule (1 × 0.6 cm) likely nodal. Fluorine-18 fluorodeoxyglucose ( ^18^ F-FDG) positron emission tomography (PET)/computed tomography (CT) was performed, which revealed a clear operative bed and FDG avid pre-tracheal and left paratracheal lymph nodes. The largest measured 1.3 × 1.6 cm, with the maximum standardized uptake value (SUVmax) of ∼16.8. ( **B** ) Pathology revealed metastatic papillary carcinoma.

In an attempt to investigate patients at higher risk of locoregional recurrence after BIR. Retrospective analyses were performed of those patients who developed pathologically proven locoregional recurrence compared with those who remained free of recurrent disease at the end of the study, to evaluate pretreatment clinicopathological factors that are associated with and perhaps predispose to the occurrence of locoregional recurrence in such patients ( Fig. 1 ).

Female patients represented 61.6% of those patients who developed locoregional recurrence ( *p* = 0.012). Fifty-seven of the 73 patients who developed recurrence had PTC, representing 78.1% of the population compared with other pathologies ( *p* = 0.001). In all, 35.6% of the patients who experienced locoregional recurrence had initial regional nodal deposits compared with 23.3% of the other group ( *p* = 0.046). For patients with locoregional recurrence, the primary tumors were T2 in 52.1% of them, T3 in 26.1%, and T1 in 21.9%, while in 55% of the patients who were negative for locoregional recurrence, the primary tumors were classified as T1 at initial staging, 27.8% as T2, and 17.2% as T3 ( *p* < 0.001).

No significant association was noted between the initial management, whether it included nodal dissection or not, and the incidence of recurrence ( *p* = 0.398). No significant age prevalence was noted among both groups ( *p* = 0.606). Vascular invasion did not reveal any significant association in both groups ( *p* = 0.636). Although no statistically significant association was found, a trend for prevalence of capsular invasion was observed among patients who developed locoregional recurrence, representing 30.1% compared with 19.4% of those who did not develop recurrence ( *p* = 0.065; Table 1 ).

**Table 1:** Pretreatment clinicopathological factors in DTC patients who developed versus those who did not develop structural recurrence after BIR (no. of patients = 253)

		Locoregional recurrence				*p* -value
		Positive		Negative		
		Number	%	Number	%	
Age	≥45 y	33	45.2	75	41.7	0.606
	< 45 y	40	54.8	105	58.3	
Sex	Female	45	61.6	139	77.2	0.012
	Male	28	38.4	41	22.8	
Pathology	PTC	57	78.1	159	88.3	0.001
	FTC	8	11.0	20	11.1	
	Hürthle cell	8	11.0	1	0.6	
Capsular Invasion	Positive	22	30.1	35	19.4	0.065
	Negative	51	69.9	145	80.6	
Vascular invasion	Positive	7	9.6	14	7.8	0.636
	Negative	66	90.4	166	92.2	
Lymph nodes metastases	Positive	26	35.6	42	23.3	0.046
	Negative	47	64.4	138	76.7	
T	T1a	3	4.1	28	15.6	< 0.001
	T1b	13	17.8	71	39.4	
	T2	38	52.1	50	27.8	
	T3a	1	1.4	6	3.3	
	T3b	18	24.7	25	13.9	
N	Positive	26	35.6	42	23.3	0.046
	Negative	47	64.4	138	76.7	
M	Positive	0	0.0	0	0.0	–
	Negative	73	100.0	180	100.0	
Initial treatment	Thyroidectomy + RAI-131 ablation	61	83.6	142	78.9	0.398
	Thyroidectomy + nodal dissection + RAI-131 ablation	12	16.4	38	21.1	

Abbreviations: BIR, biochemical incomplete response; DTC, differentiated thyroid cancer; FTC, follicular thyroid carcinoma; PTC, papillary thyroid carcinoma.

### Thyroglobulin Course and Structural Locoregional Recurrence

At the first 6 months of follow-up after RAI-131 ablation, the entire group showed an incomplete biochemical response as the median value for Tg was 17 (range, 10–93) ng/mL, and on further 1-year follow-up, the median value of Tg level for the entire group was 5.7 (range, 0.2–220) ng/mL.

The median value of Tg level for the group who later on developed locoregional recurrence compared with those who remained free at the last follow-up was 18 (range, 13–93) versus 17 (range, 10–90) ng/mL. On further 1-year follow-up, the median value spontaneously increased for the positive group to 37.6 (range, 15–220) ng/mL and decreased for those who remained free to 3 (range, 0.2 - 58) ng/mL.

The median time to recurrence was 27 months. The median size of the operative bed lesions was 2.2 (range, 1–5) cm, the median value of their SUVmax was 7.4 (range, 3.5–15.2). The median size of the regional lymph nodes was 1.7 (range, 0.8–8.8) cm, and the median value of their SUVmax was 8.1 (range, 2.9–19.4).

By evaluating the course of the Tg level over a 1-year follow-up in relation to various clinicopathological parameters, 78.6% of the patients aged less than 45 years showed declining Tg levels compared with 63.9% of those ≥45 years ( *p* = 0.017). In all, 37.7% of males revealed progressive elevation of the Tg level compared with 22.8% of females ( *p* = 0.043). In total, 88.9% of patients with Hürthle cell tumors had rising Tg levels compared with 24.5 and 25.0% of those with PTC and FTC, respectively ( *p* < 0.001). Among patients who showed a progressive Tg course, 73.6% were classified as T2 and T3, while 52% of those with a declining course were classified as T1 ( *p* = 0.005). Eighty percent of the patients with a rising Tg level developed structural locoregional recurrence compared with 7.7% of those with a declining course ( *p* < 0.001). One-hundred percent of those with declining and stable Tg levels showed complete response to therapy compared with 70.6% of those with a rising Tg level ( *p* < 0.001). Among patients with a rising Tg level, 11.8% died on follow-up compared with 2.7 and 0% of patients with a declining and stable course ( *p* = 0.019). No significant correlation was observed between capsular invasion, vascular invasion, N-stage, primary treatment, and time to recurrence in respect to the course of Tg ( Table 2 ).

**Table 2:** Course of Tg level over a 1-year follow-up in relation to various clinicopathological parameters (no. of patients = 253)

**Clinicopathological**	**Tg course**			***p*** **-value**
	**Stable**	**Elevating**	**Declining**	
**Age**				
≥45 y	1 (50.0%)	38 (55.9%)	69 (37.7%)	0.017
< 45 y	1 (50.0%)	30 (44.1%)	114 (62.3%)	
**Sex**				
Female	2 (100.0%)	42 (61.8%)	140 (76.5%)	0.043
Male	0 (0.0%)	26 (38.2%)	43 (23.5%)	
**Pathological type**				
PTC	1 (50.0%)	53 (77.9%)	162 (88.5%)	< 0.001
FTC	1 (50.0%)	7 (10.3%)	20 (10.9%)	
Hürthle cell	0 (0.0%)	8 (11.8%)	1 (0.55%)	
**Capsular invasion**				
Positive	1 (50.0%)	19 (27.9%)	37 (20.2%)	0.185
Negative	1 (50.0%)	49 (72.0%)	146 (79.8%)	
**Vascular invasion**				
Positive	0 (0.0%)	7 (10.3%)	14 (7.7%)	0.670
Negative	2 (100.0%)	61 (89.7%)	169 (92.3%)	
**T stage**				
T1a	0 (0.0%)	4(5.8%)	27 (14.8%)	0.005
T1b	1 (50.0%)	14 (20.6%)	69 (37.7%)	
T2	0 (0.0%)	34 (50.0%)	54 (29.5%)	
T3a	0 (0.0%)	1 (1.5%)	6 (3.2%)	
T3b	1 (50.0%)	15 (22.1%)	27 (14.8%)	
**N stage**				
N0	1 (50.0%)	45 (24.3%)	139 (76.0%)	0.173
N1	1 (50.0%)	23 (33.8%)	44 (24.0%)	
**Initial treatment**				
Thyroidectomy + RAI-131 ablation	1 (50.0%)	57 (28.1%)	145 (79.2%)	0.289
Thyroidectomy + nodal dissection + RAI-131 ablation	1 (50.0%)	11 (22.0%)	38 (20.8%)	
**Locoregional recurrence**				
Positive	0 (0.0%)	59 (80.8%)	14 (7.7%)	< 0.001
Negative	2 (100.0%)	9 (5.0%)	169 (92.3%)	
**Time to recurrence**				
≤23 mo	0 (0.0%)	33 (55.9%)	4 (28.6%)	0.150
> 23 mo	0 (0.0%)	26 (44.1%)	10 (71.4%)	
**Response**				
Complete response	2 (100.0%)	48 (70.6%)	183 (100.0%)	< 0.001
Incomplete response	0 (0.0%)	20 (29.4%)	0 (0.0%)	
**Mortality**				
Alive	2 (100.0%)	60 (88.2%)	178 (97.3%)	0.019
Dead	0 (0.0%)	8 (11.8%)	5 (2.7%)	

Abbreviations: FTC, follicular thyroid carcinoma; PTC, papillary thyroid carcinoma.

As a trial to investigate the impact of 6 months of postablation Tg and 1-year follow-up Tg levels on disease outcome, the ROC curve was first tested to mark the cutoff point with compromised sensitivity and specificity for postablation Tg (6 months) and 1-year follow-up Tg, to discriminate positive versus negative locoregional recurrence; however, such cutoff points could not be obtained ( *p* > 0.05). Thus, their median values have been used instead as cutoffs and for correlations in respect to different clinicopathological parameters. The 6-month postablation Tg did not reveal any significant correlations with the occurrence of locoregional recurrence, treatment response, time to recurrence, survival, or occurrence of distant metastases ( Table 3 ). Meanwhile assessing the median value of the 1-year follow-up stimulated Tg revealed significant correlations with the occurrence of locoregional recurrence. Among those who remained recurrence free, 91.3% had a Tg level lower than 5.7 ng/mL compared to only 8.7% who developed locoregional recurrence ( *p* < 0.001). One-hundred percent of those with Tg ≤ 5.7 ng/mL showed complete therapy response compared with 84.1% of those with Tg > 5.7 ng/mL ( *p* < 0.001). Ten out of the 13 patients who died on follow-up had T9 > 5.7 ng/mL ( *p* = 0.045). No significant correlations were obtained in relation to time to recurrence or the occurrence of distant metastases ( *p* = 0.761 and 1) respectively ( Table 4 ).

**Table 3:** The median value of the 6 months post ablation Tg level of the group (17) was assessed in respect different clinicopathological parameters

Outcome		6 months Post-ablation Tg				*p* -value
		≤ 17		> 17		
		Number	%	Number	%	
Loco-regional recurrence	Positive	35	27.1	38	30.6	0.633
	Negative	94	72.9	86	69.4	
Complete/incomplete response	Complete	120	93.0	113	91.1	0.577
	Incomplete	9	7.0	11	8.9
Time to recurrence (mo)	≤23	18	48.6	20	51.3	0.818
	> 23	19	51.4	19	48.7	
Survival	Alive	124	96.1	116	93.5	0.354
	Died	5	3.9	8	6.5	
Distant metastases	Positive	2	1.6	5	4.0	0.274
	Negative	127	98.4	119	96.0	

**Table 4:** The median value of the 1-year follow-up Tg level of the group (5.7) was assessed in respect different clinicopathological parameters.

Outcome		1-year follow-up Tg				*p* -value
		≤5.7		> 5.7		
		Number	%	Number	%	
Locoregional recurrence	Positive	11	8.7	62	49.2	< 0.001
	Negative	116	91.3	64	50.8	
Complete/incomplete response	Complete	127	100.0	106	84.1	< 0.001
	Incomplete	0	0.0	20	15.9	
Time to recurrence (mo)	≤23	6	46.2	32	50.8	0.761
	> 23	7	53.8	31	49.2	
Survival	Alive	124	97.6	116	92.1	0.045
	Died	3	2.4	10	7.9	
Distant metastases	Positive	4	3.1	3	2.4	1
	Negative	123	96.9	123	97.6	

### ^18^ F-FDG PET/CT versus Other Standard Techniques Modalities on Diagnostic and Prognostic Basis

With regard to the diagnostic performance of ^18^ F-FDG PET/CT in the detection of structural locoregional recurrence in comparison to other standard techniques, ^18^ F-FDG PET/CT revealed the highest sensitivity and NPV of 100% compared to sensitivity of 92.86 and 85.71% and NPV of 99.58 and 99.17%, respectively, with US and RAI-131 scan. Meanwhile, the highest specificity and PPV (100%) were observed with RAI-131 compared to specificity values of 99.16 and 99.58% and PPV of 87.50 and 92.86%, respectively, with ^18^ F-FDG PET/CT and US ( Table 5 ).

**Table 5:** Diagnostic performance of ^18^ F-FDG PET/CT versus US versus RAI-131 in detection of structural locoregional recurrence in DTC patients

Performance	PET/CT	Neck U/S	RAI-131
Sensitivity	100.00% 95% CI (76.84–100.00%)	92.86% 95% CI (66.13–99.82%)	85.71% 95% CI (57.19–98.22%)
Specificity	99.16% 95% CI (97.01–99.90%)	99.58% 95% CI (97.69–99.99%)	100.00% 95% CI (98.47–100.0%)
PPV	87.50% 95% CI (63.78–96.53%)	92.86% 95% CI (64.65–98.93%)	100.00% 95% CI (73.54–100.0%)
NPV	100.00% 95% CI (98.46–100.00%)	99.58% 95% CI (97.30–99.94%)	99.17% 95% CI (97.07–99.77%)
Accuracy	99.21% 95% CI (97.17–99.90%)	99.21% 95% CI (97.17–99.90%)	99.21% 95% CI (97.17–99.90%)

Abbreviations: CI, confidence interval; CT, computed tomography; DTC, differentiated thyroid cancer; ^18^ F-FDG, fluorine-18 fluorodeoxyglucose; NPV, negative predictive value; PET, positron emission tomography; PPV, positive predictive value; US, ultrasound.

On per nodal analysis, a higher number of lymph nodes were detected by ^18^ F-FDG PET/CT compared with US at different levels where ^18^ F-FDG PET/CT exceeded the US in the total number of nodes detected by 40 lymph nodes. The highest difference was at level VI, followed by level IV, and the least difference was at level I ( Table 6 ).

**Table 6:** Number of lymph nodes detected by ^18^ F-FDG PET/CT versus neck US at different levels

Lymph nodes level	Number of lymph nodes detected	
	^18^ F-FDG PET/CT	US
Level I	6	5
Level II	25	22
Level III	19	14
Level IV	32	25
Level V	10	6
Level VI	51	35
Supraclavicular LNs	8	4
Total number of lymph nodes	151	111

Abbreviations: CT, computed tomography; ^18^ F-FDG, fluorine-18 fluorodeoxyglucose; LN, lymph node; PET, positron emission tomography; US, ultrasound.

### Predictive Value of ^18^ F-FDG PET/CT

**Fig. 2:**
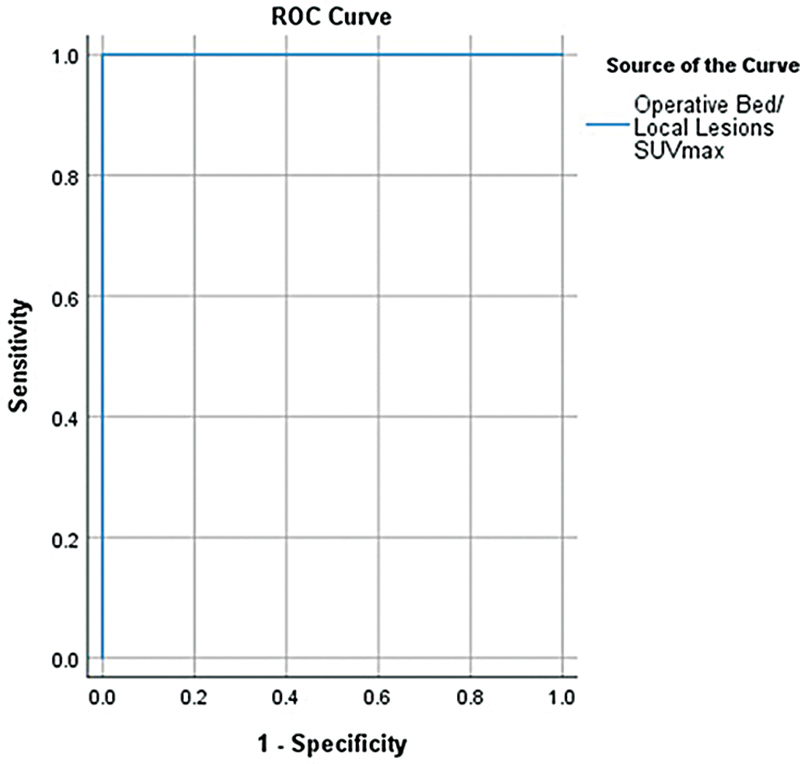
Receiver operating characteristic (ROC) curve to test the maximum standardized uptake value (SUVmax) capability to differentiate differentiated thyroid cancer patients who were positive versus negative for structural local recurrence ( *p* < 0.001).

The ROC curve was tested to mark the cutoff point for SUVmax with compromised sensitivity and specificity to discriminate patients with positive versus negative structural local recurrence, complete response versus incomplete response, long versus short time to recurrence (using the median value as a cutoff), and survival (alive versus dead). A cutoff point (SUVmax = 3.65) was successfully marked to discriminate positive versus negative local recurrence with sensitivity and specificity of 100% and *p* -value less than 0.001. Other cutoff points, in respect to response, time to recurrence, and survival, could not be obtained ( *p* > 0.05; Fig. 2 ; Table 7 ).

**Table 7:** Value of SUVmax as a discriminator in respect to structural local recurrence, response; time to recurrence and Survival in patients with DTC

End point	SUVmax						
	AUC	*p* -value	95% confidence interval		Cutoff	Sensitivity (%)	Specificity (%)
			Lower bound	Upper bound			
Local recurrence	1.000	< 0.001	1.000	1.000	3.65	100	100
Response	0.545	0.787	0.216	0.875	–	–	–
Time to recurrence	0.520	0.901	0.199	0.841	–	–	–
Survival	0.417	0.696	-0.001-	0.834	–	–	–

Abbreviations: AUC, area under the curve; DTC, differentiated thyroid cancer; SUVmax, maximum standardized uptake value.

## Discussion

BIR in DTC is one of the most challenging events that may occur post radio-surgical treatment with still unclear predisposing factors and concerns about its outcomes. 7 Since Tg level is a cornerstone parameter in follow-up of treated DTC for early detection of structural recurrence, its failure to decline to undetectable level postthyroidectomy and RAI-131 ablation makes the follow-up problematic. This raises concerns for microscopic disease that remains beyond radiological detectability. 18 In clinical practice, BIR does not seem to be restricted to only patients initially classified as high risk; hence, few studies attempted to explore specific risk factors that may be linked to BIR. Lim et al aimed at constructing a nomogram to determine the predictors of BIR. 19 Their multivariate analysis demonstrated that age ≥55 years, male sex, site of lymph node metastasis, presence of extrathyroidal extension, and presence of lymphovascular invasion were significantly associated with BIR. In our group, 72.7% of patients who experienced BIR were females, 42.7% were ≥45 years, and 26.9% were classified as N1. Capsular invasion was positive in 22.5% and vascular invasion was positive in only 8.3% patients. PTC was found in 85.4% of the group, the median size of the primary tumor was 2.4 cm, 45.45% patients were classified as T1, and all were M0.

One of the major prognostic concerns is whether BIR predisposes to higher incidence of recurrence; thus, such patients are usually exposed to unnecessary excessive management. 8 In published literature, only few studies investigated the poor prognostic factors in DTC patients with BIR with a relatively small number of enrolled patients. The number of patients who developed structural recurrent disease was also low. 20 21 22 23 24 In the current study, 253 patients with BIR were enrolled, among which 73 patients (28.85%) developed structural locoregional recurrence with a median time to recurrence of 27 months. Previous studies have reported that 5 to 27% of patients with DTCs develop locoregional recurrences. 25 Such recurrences have been reported to be located in cervical lymph nodes in 60 to 75% of cases and thyroid bed in approximately 20% of cases, worsening the prognosis and leading to a risk of cancer-related death. 25 A slightly higher incidence is noted among our group due to the already present posttreatment elevated Tg level as a risk factor. In another study performed by Ahn et al, they evaluated the long-term outcomes of 102 patients with PTC showing a BIR during the first 12 to 24 months following initial therapy. Structural persistent disease was observed in 43 (42%) patients. 24

An attempt was performed in the present study to further explore those patients at higher risk of structural locoregional recurrence after BIR. Our enrolled population revealed a predominance for female patients (61.6%) among those who developed structural locoregional recurrence; however, the higher incidence of DTC among females and the high number of the female patients already enrolled within the study population can stand behind such predominance. PTC is known for being the most familiar type of thyroid carcinoma, accounting for nearly 90% of all thyroid carcinomas with early dissemination to regional lymph nodes where the postoperative serum Tg is one of its prognostic indicators. 26 In the present study, PTC represented 78.1% of those who developed locoregional recurrence compared with other pathologies ( *p* = 0.001). A higher number (35.6%) of such patients also had initial regional nodal deposits compared with 23.3% of those who remained free from locoregional recurrence till the last follow-up ( *p* = 0.046). For patients developed locoregional recurrence; their primary tumors were of higher T stages compared with those who were negative for locoregional recurrence ( *p* < 0.001). However, age, vascular invasion, and initial management did not reveal a significant association ( *p* > 0.05). A trend for the prevalence of capsular invasion was observed among patients who developed locoregional recurrence, representing 30.1% compared with 19.4% of those who did not develop recurrence ( *p* = 0.065).

Current trends in clinical practice adopted DRA in DTC; thus, not only the post–radio-surgical ablation level of Tg matters but also its course overtime should be considered the key parameters for dynamic risk stratification in DTC. 2 At the first 6 months of follow-up after RAI-131 ablation, the entire study group showed an incomplete biochemical response; the median value for stimulated Tg was 17 (range, 10–93), and on further 1-year follow-up, the median value of stimulated Tg level for the entire group was 5.7 (range, 0.2–220). At the initial 6 months of follow-up, no significant difference in respect to the median value of the Tg level for the group who later on developed locoregional recurrence compared with those who remained free at the last follow-up was observed (18 vs. 17, respectively). However, a gap between both groups appeared on further 1-year follow-up, where the median value spontaneously increased for the positive group to 37.6 and decreased for those who remained free to 3. Also, the majority (80%) of the patients with rising Tg level developed structural locoregional recurrence compared with only 7.7% of those with a declining course ( *p* <0.001). Such results matched those of Wang et al, who concluded that the incremental course of posttreatment Tg is a significant predictor for structural recurrent/persistent disease with high PPV of 81% when it exceeds 20.2 ng/mL, which provides a prompt identification of those at higher risk and paves the way to enable subsequent intensive management and follow-up. 7 Also, several studies revealed a strong association between rising TG level and recurrence in DTC. 27 28 29

By evaluating the course of the posttreatment stimulated Tg level over a 1-year follow-up in relation to various clinicopathological parameters, older age (≥45 years), male sex, Hürthle cell tumors, and T2 and T3 primary tumors revealed progressive elevation of the Tg level compared with other parameters ( *p* < 0.05). A higher number (29.4%) of patients showed a rising Tg course had incomplete therapy response at last follow-up, while all patients with a declining and stable Tg level showed complete response ( *p* < 0.001) and 11.8% of them with a rising Tg died on follow-up compared with 2.7 and 0% of a declining and stable course ( *p* = 0.019). But no significant correlation between capsular invasion, vascular invasion, N-stage, primary treatment, and time to recurrence in respect to the course of Tg was observed.

In respect to the median Tg value at the initial 6-month versus 1-year follow-up, the former did not significantly correlate with risk of locoregional recurrence or any different clinicopathological factors. On the contrary, longer follow-up at the 1-year median Tg value revealed significant correlations between locoregional recurrence, therapy response, and mortality ( *p* <0.05). No significant correlations were also obtained with time to recurrence or the occurrence of distant metastases ( *p* = 0.761 and 1, respectively).

As previously mentioned, the main seriousness of BIR stands behind the risk of developing structural disease that is detected by morphological imaging and calling for a strategic therapeutic approach. 23 Standard diagnostic imaging techniques in DTC are neck US, CT, magnetic resonance imaging (MRI), and nuclear imaging, such as postdiagnostic or therapeutic radioiodine whole-body scan and/or ^18^ F-FDG PET/CT. 30 31 US is cost-effective and accessible for detecting cervical recurrence, but it is not without limitations such as false-positive results. 32 RAI imaging is crucial for RAI-avid disease but is ineffective in RAI-refractory cases. ^18^ F-FDG PET/CT is cost-effective and indispensable in high-risk or RAI-refractory disease but is limited by its high cost and availability. 33 Therefore, a more widely adopted stepwise approach, using US and RAI 131 as first-line tools, and reserving ^18^ F-FDG PET/CT for high-risk or complex cases, offers an optimal balance between cost-effectiveness and accessibility in clinical practice.

The role of ^18^ F-FDG PET/CT in DTC has been specifically highlighted in the cases with rising Tg and negative radioactive iodine WBS. Meanwhile, ^18^ F-FDG PET/CT is not only a noninvasive whole-body screening tool but also may reflect disease aggressiveness and provide information about the long-term outcome, which might influence and even modify the therapeutic approach in some patients. 31 There were controversial data about the impact of TSH stimulation on the accuracy of ^18^ F-FDG PET/CT, with the clinical benefit not clearly identified. 34 With regard to Tg, although there is no consensus on the cutoff value that provides the optimum diagnostic accuracy of ^18^ F-FDG PET/CT to detect local and/or distant disease recurrence, the ATA guidelines recommend that a stimulated Tg level greater 10 ng/mL should be an adequate indicator. 35 In the current study, the diagnostic performance of ^18^ F-FDG PET/CT in the detection of structural locoregional recurrence was 100, 99.16, and 87.50% in terms of sensitivity, specificity, and PPV, respectively. Abelleira et al 36 reported a sensitivity of 95% and a specificity of 87.5%, while Lu et al 37 reported a sensitivity and PPV of 93.30 and 91.40%, respectively. In respect to other standard techniques, the highest sensitivity and NPV of 100% were obtained by ^18^ F-FDG PET/CT compared to sensitivity values of 92.86 and 85.71% and NPV of 99.58 and 99.17%, respectively, with US and RAI-131 scan, while the highest specificity and PPV (100%) were observed with RAI-131 compared to specificity values of 99.16 and 99.58% and PPV of 87.50 and 92.86%, respectively, with ^18^ F-FDG PET/CT and US. The median size of the operative bed lesions was 2.2 cm, the median value of their SUVmax was 7.4, the median size of the regional lymph nodes was 1.7, and the median value of their SUVmax was 8.1. A higher number of regional lymph nodes were detected by ^18^ F-FDG PET/CT compared to US and was specially noted at level VI, followed by level IV, and the least difference was at level I.

Quantitation is one of the main advantages of PET/CT. A predefined SUVmax cutoff value can help in differentiating malignant lesions from benign ones, improving diagnostic accuracy. High SUVmax lesions might indicate a more aggressive phenotype and may predict RAI refractory disease, guiding a shift toward alternative therapies. 38 However, there is still lack of standardization of the SUVmax cutoffs with potential limitation, as there may be false-positive or false-negative results and its role still complementary. In the present study, a cutoff point (SUVmax 3.65) was successfully marked to discriminate positive versus negative local recurrence with specificity of 100% and *p* -value less than 0.001. A close cutoff point of SUVmax 3.2 was found to be ideal to detect structural disease by Abelleira et al with a specificity of 95%. 36

With regard to the prognostic role of ^18^ F-FDG PET/CT, several previous studies reported PET/CT parameters as promising prognostic markers in terms of disease progression and survival in patients with DTC. Pace et al 39 demonstrated that patients with no FDG avidity in FDG PET/CT scans have better progression-free survival whether in the whole group or in those with elevated TG. Salvatore et al 40 reported that Tg normalization and ^18^ F-FDG PET/CT were independent predictors of disease-free survival at short-term follow-up. Gaertner et al 41 concluded that FDG PET in high-risk DTC patients is predictive for survival when performed at the time of thyroid remnant ablation. In the current study, the ROC curve analysis revealed no cutoff points could be determined to predict the response to therapy or survival, likely due to heterogeneity of the enrolled risk groups.

This study has some limitations due to its retrospective design, which carries certain inherent limitations such as selection bias, as the data were collected from preexisting records. Also, relying on the retrieved data where stimulated Tg levels for all enrolled patients was available but unstimulated Tg and Tg Ab were not available for the entire population and therefore were not evaluated in the current study. This might influence the applicability of the findings. However, a strong point of the study is that to the best of our knowledge, the current study enrolled the largest number of DTC patients with BIR with comprehensive clinicopathological, laboratory, and imaging assessment.

## Conclusion

BIR is a challenging event after radio-surgical treatment of DTC patients with no distinct consensus for management. In the current study, a considerable rate of structural locoregional recurrence was noted in such patients and was more commonly associating worse clinicopathological parameters. Tg is a core parameter for DRA in DTC, and its incremental levels carry the worse prognosis in BIR patients and seems to be linked to initial poor clinicopathological parameters and indicate higher incidence of structural disease recurrence. In the current study, ^18^ F-FDG PET/CT demonstrated a valuable diagnostic role rather than prognostic role for structural disease recurrence in BIR patients.

## References

[1] Kim T Y Kim W G Kim W B Shong Y K Current status and future perspectives in differentiated thyroid cancer Endocrinol Metab (Seoul) 2014 29 03 217 225 25309778 10.3803/EnM.2014.29.3.217 PMC4192824

[2] Pitoia F Jerkovich F Dynamic risk assessment in patients with differentiated thyroid cancer Endocr Relat Cancer 2019 26 10 R553 R566 31394499 10.1530/ERC-19-0213

[3] Tuttle R M Alzahrani A S Risk stratification in differentiated thyroid cancer: from detection to final follow-up J Clin Endocrinol Metab 2019 104 09 4087 4100 30874735 10.1210/jc.2019-00177 PMC6684308

[4] American Thyroid Association (ATA) Guidelines Taskforce on Thyroid Nodules and Differentiated Thyroid Cancer Cooper D S Doherty G M Haugen B R Revised American Thyroid Association management guidelines for patients with thyroid nodules and differentiated thyroid cancer Thyroid 2009 19 11 1167 1214 19860577 10.1089/thy.2009.0110

[5] Pitoia F Ward L Wohllk N Recommendations of the Latin American Thyroid Society on diagnosis and management of differentiated thyroid cancer Arq Bras Endocrinol Metabol 2009 53 07 884 887 19942992 10.1590/s0004-27302009000700014

[6] Luster M Aktolun C Amendoeira I European perspective on 2015 American Thyroid Association management guidelines for adult patients with thyroid nodules and differentiated thyroid cancer: proceedings of an interactive international symposium Thyroid 2019 29 01 7 26 30484394 10.1089/thy.2017.0129

[7] Wang Y Wu J Jiang L Zhang X Liu B Prognostic value of post-ablation stimulated thyroglobulin in differentiated thyroid cancer patients with biochemical incomplete response: a bi-center observational study Endocrine 2022 76 01 109 115 35094313 10.1007/s12020-021-02976-8

[8] Tuttle R M Optimal management of a biochemical incomplete response to therapy in differentiated thyroid cancer: aggressive treatment or cautious observation? Endocrine 2014 46 03 363 364 24615658 10.1007/s12020-014-0213-2

[9] Kukulska A Krajewska J Kołosza Z The role of FDG-PET in localization of recurrent lesions of differentiated thyroid cancer (DTC) in patients with asymptomatic hyperthyroglobulinemia in a real clinical practice Eur J Endocrinol 2016 175 05 379 385 27511823 10.1530/EJE-16-0360

[10] Kim B H Kim S J Kim K High metabolic tumor volume and total lesion glycolysis are associated with lateral lymph node metastasis in patients with incidentally detected thyroid carcinoma Ann Nucl Med 2015 29 08 721 729 26108700 10.1007/s12149-015-0994-2 PMC4661206

[11] Palmedo H Bucerius J Joe A Integrated PET/CT in differentiated thyroid cancer: diagnostic accuracy and impact on patient management J Nucl Med 2006 47 04 616 624 16595495

[12] Shah J P Montero P H New AJCC/UICC staging system for head and neck, and thyroid cancer Rev Med Clin Las Condes 2018 29 04 397 404

[13] Haugen B R Alexander E K Bible K C 2015 American Thyroid Association management guidelines for adult patients with thyroid nodules and differentiated thyroid cancer: the American Thyroid Association guidelines task force on thyroid nodules and differentiated thyroid cancer Thyroid 2016 26 01 1 133 26462967 10.1089/thy.2015.0020 PMC4739132

[14] European Association of Nuclear Medicine (EANM) Boellaard R Delgado-Bolton R Oyen W J FDG PET/CT: EANM procedure guidelines for tumour imaging: version 2.0 Eur J Nucl Med Mol Imaging 2015 42 02 328 354 25452219 10.1007/s00259-014-2961-x PMC4315529

[15] Chan Y H Biostatistics 102: quantitative data—parametric & non-parametric tests Singapore Med J 2003 44 08 391 396 14700417

[16] Chan Y H Biostatistics 103: qualitative data: tests of independence Singapore Med J 2003 44 10 498 503 15024452

[17] Galen R S Predictive value and efficiency of laboratory testing Pediatr Clin North Am 1980 27 04 861 869 7454413 10.1016/s0031-3955(16)33930-x

[18] Phan H T Jager P L van der Wal J E The follow-up of patients with differentiated thyroid cancer and undetectable thyroglobulin (Tg) and Tg antibodies during ablation Eur J Endocrinol 2008 158 01 77 83 18166820 10.1530/EJE-07-0399

[19] Lim S T Jeon Y W Gwak H Bae J S Suh Y J Nomogram for the prediction of biochemical incomplete response in papillary thyroid cancer patients Cancer Manag Res 2021 13 5641 5650 34285584 10.2147/CMAR.S320993 PMC8286100

[20] Vaisman F Tala H Grewal R Tuttle R M In differentiated thyroid cancer, an incomplete structural response to therapy is associated with significantly worse clinical outcomes than only an incomplete thyroglobulin response Thyroid 2011 21 12 1317 1322 22136267 10.1089/thy.2011.0232

[21] Vaisman F Momesso D Bulzico D A Spontaneous remission in thyroid cancer patients after biochemical incomplete response to initial therapy Clin Endocrinol (Oxf) 2012 77 01 132 138 22248037 10.1111/j.1365-2265.2012.04342.x

[22] Pitoia F Abelleira E Tala H Bueno F Urciuoli C Cross G Biochemical persistence in thyroid cancer: is there anything to worry about? Endocrine 2014 46 03 532 537 24287799 10.1007/s12020-013-0097-6

[23] Zern N K Clifton-Bligh R Gill A J Disease progression in papillary thyroid cancer with biochemical incomplete response to initial therapy Ann Surg Oncol 2017 24 09 2611 2616 28585075 10.1245/s10434-017-5911-6

[24] Ahn J Song E Kim W G Long-term clinical outcomes of papillary thyroid carcinoma patients with biochemical incomplete response Endocrine 2020 67 03 623 629 31776976 10.1007/s12020-019-02142-1

[25] Rouxel A Hejblum G Bernier M O Prognostic factors associated with the survival of patients developing loco-regional recurrences of differentiated thyroid carcinomas J Clin Endocrinol Metab 2004 89 11 5362 5368 15531482 10.1210/jc.2003-032004

[26] Arrangoiz R De Llano J G Mijares M F Current understanding of papillary thyroid carcinoma Int J Otolaryngol Head Neck Surg 2021 10 03 184 221

[27] Yim J H Kim E Y Bae Kim W Long-term consequence of elevated thyroglobulin in differentiated thyroid cancer Thyroid 2013 23 01 58 63 22973946 10.1089/thy.2011.0487 PMC3539255

[28] Duren M Siperstein A E Shen W Duh Q Y Morita E Clark O H Value of stimulated serum thyroglobulin levels for detecting persistent or recurrent differentiated thyroid cancer in high-and low-risk patients Surgery 1999 126 01 13 19 10418587 10.1067/msy.1999.98849

[29] Carvalho J MG Görlich D Schober O Evaluation of 131 I scintigraphy and stimulated thyroglobulin levels in the follow up of patients with DTC: a retrospective analysis of 1420 patients Eur J Nucl Med Mol Imaging 2017 44 744 756 27909769 10.1007/s00259-016-3581-4

[30] Agate L Bianchi F Giorgetti A Detection of metastases from differentiated thyroid cancer by different imaging techniques (neck ultrasound, computed tomography and [ ^18^ F]-FDG positron emission tomography) in patients with negative post-therapeutic ^131^ I whole-body scan and detectable serum thyroglobulin levels J Endocrinol Invest 2014 37 10 967 972 25070043 10.1007/s40618-014-0134-1

[31] Shalash A M Elahmadawy M A Heikal S Y Amin A A Youssef A A Value of diffusion MRI versus [ ^18^ F]FDG PET/CT in detection of cervical nodal metastases in differentiated thyroid cancer patients Nucl Med Rev Cent East Eur 2022 25 02 112 118 35971948 10.5603/NMR.a2022.0035

[32] Lamartina L Deandreis D Durante C Filetti S Endocrine tumours: imaging in the follow-up of differentiated thyroid cancer: current evidence and future perspectives for a risk-adapted approach Eur J Endocrinol 2016 175 05 R185 R202 27252484 10.1530/EJE-16-0088

[33] Campennì A Barbaro D Guzzo M Capoccetti F Giovanella L Personalized management of differentiated thyroid cancer in real life: practical guidance from a multidisciplinary panel of experts Endocrine 2020 70 02 280 291 32772339 10.1007/s12020-020-02418-x PMC7581611

[34] Qichang W Lin B Gege Z Diagnostic performance of ^18^ F-FDG-PET/CT in DTC patients with thyroglobulin elevation and negative iodine scintigraphy: a meta-analysis Eur J Endocrinol 2019 181 02 93 102 31117054 10.1530/EJE-19-0261

[35] Smallridge R C Diehl N Bernet V Practice trends in patients with persistent detectable thyroglobulin and negative diagnostic radioiodine whole body scans: a survey of American Thyroid Association members Thyroid 2014 24 10 1501 1507 25058708 10.1089/thy.2014.0043 PMC4195231

[36] Abelleira E García Falcone M G Bueno F Pitoia F Role of ^18^ F-FDG-PET/CT in patients with differentiated thyroid cancer with biochemical incomplete or indeterminate response to treatment Endocrinol Diabetes Nutr (Engl Ed) 2020 67 08 517 524 32534971 10.1016/j.endinu.2020.02.007

[37] Lu C Z Cao S S Wang W Liu J Fu N Lu F Usefulness of PET/CT in the diagnosis of recurrent or metastasized differentiated thyroid carcinoma Oncol Lett 2016 11 04 2420 2423 27073490 10.3892/ol.2016.4229 PMC4812582

[38] Tang X Shi L Zhao Z Clinical role of ^18^ F-FDG PET/CT for detection of radioactive iodine refractory differentiated thyroid cancer Medicine (Baltimore) 2023 102 24 e33878 37327310 10.1097/MD.0000000000033878 PMC10270557

[39] Pace L Klain M Salvatore B Prognostic role of ^18^ F-FDG PET/CT in the postoperative evaluation of differentiated thyroid cancer patients Clin Nucl Med 2015 40 02 111 115 25546215 10.1097/RLU.0000000000000621

[40] Salvatore B Klain M Nicolai E Prognostic role of FDG PET/CT in patients with differentiated thyroid cancer treated with 131-iodine empiric therapy Medicine (Baltimore) 2017 96 42 e8344 29049252 10.1097/MD.0000000000008344 PMC5662418

[41] Gaertner F C Okamoto S Shiga T FDG PET performed at thyroid remnant ablation has a higher predictive value for long-term survival of high-risk patients with well-differentiated thyroid cancer than radioiodine uptake Clin Nucl Med 2015 40 05 378 383 25608175 10.1097/RLU.0000000000000699

